# *Nostoc sphaeroids* Kütz ameliorates hyperlipidemia and maintains the intestinal barrier and gut microbiota composition of high‐fat diet mice

**DOI:** 10.1002/fsn3.1521

**Published:** 2020-04-14

**Authors:** Fenfen Wei, Yinlu Liu, Cuicui Bi, Sheng Chen, Yulan Wang, Bo Zhang

**Affiliations:** ^1^ Research Institute for Science and Technology of Functional Foods Beijing Union University Beijing China; ^2^ Beijing Key Laboratory of Bioactive Substances and Functional Foods Beijing Union University Beijing China; ^3^ Hunan Yandi Bioengineering Co., Ltd. Zhuzhou China

**Keywords:** gut microbiota, hyperlipidemia, inflammation, *Nostoc sphaeroids* Kütz, short‐chain acids

## Abstract

Hyperlipidemia is associated with chronic inflammation and intestinal dysbiosis. The purpose of this study was to investigate the protective effect of *Nostoc sphaeroids* Kütz (NO) on diet‐induced hyperlipidemia in mice. Experimental animals received a high‐fat diet (HFD) for 4 weeks, then an HFD supplemented with 2.5% or 7.5% NO for 6 weeks. HFD‐fed mice exhibited a significant increase in serum total cholesterol, triglycerides, and low‐density lipid cholesterol, and a decrease in high‐density lipid cholesterol. NO supplementation was associated with significantly lower dyslipidemia, decreased intestinal inflammation, and inhibition of toll‐like receptor 4 gene repression in HFD‐fed mice. Results suggest that NO treatment protected the integrity of the intestinal barrier. NO treatment was also associated with significant changes in the intestinal microbiota induced by HFD and an increase in the Firmicutes‐to‐Bacteroidetes ratio. Furthermore, NO treatment was also inversely correlated with mice obesity and hyperlipidemia NO and was associated with no significant in fecal short‐chain fatty acids. In conclusion, NO significantly ameliorated hyperlipidemia induced by a HFD in mice, potentially via a decrease intestinal inflammation, increase in intestinal barrier integrity, and amelioration in the gut microbiota.

## Graphical abstract

*Nostoc sphaeroids* Kütz ameliorates hyperlipidemia. *Nostoc sphaeroids* Kütz protects intestinal barrier. *Nostoc sphaeroids* Kütz ameliorates intestinal barrier.

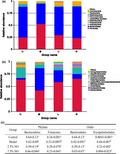

## 1 INTRODUCTION

Hyperlipidemia is a symptom associated with numerous health problems and reduced life expectancy (Bin‐Jumah, 2018; Horton, Goldstein, & Brown, 2002). There is increasing evidence that hyperlipidemia is closely linked to chronic inflammation, which can lead to cardiovascular disease, fatty liver disease, and type 2 diabetes (Feuerer et al., 2009; Hoyt, Burnette, & Auster‐Gussman, 2014). Altered gut microbiota composition has been linked to the development of hyperlipidemia and cardiometabolic diseases (Chan et al., 2016). A healthy intestinal environment comprises host and microbial factors, and relies on a functionally intact intestinal barrier and stable microbiota composition (Lu et al., 2016; Monk et al., 2019; Ojo et al., 2016). However, studies suggest that high‐fat diets (HFDs) may cause gut microbiota dysbiosis and intestinal integrity destruction. This process may cause an increase in blood lipopolysaccharide (LPS) concentration and was shown to increase inflammation in mice due to the activation of toll‐like receptor 4 (TLR4)‐mediated inflammation (Perry et al., 2016; Rooks & Garrett, 2016; Schroeder & Backhed, 2016). These findings indicate that HFD‐induced hyperlipidemia in mice could be reversed by restoring the gut microbiota and intestinal barrier.

The available drugs for hyperlipidemia treatment include statins and fibrates, but these have side effects and are expensive, and have shown very limited effectiveness (Bin‐Jumah, 2018; De Leo et al., 2005; Lee, Chun, Kwon, Kim, & Nam, 2017). Many researchers have also investigated and developed substances with lipid‐lowering functions from natural products. The habitual consumption of foods with antilipid metabolism disorder effects may be an effective and manageable way to prevent hyperlipidemia.

Blue‐green algae (BGA) are one of earth's most primitive life forms and have been utilized in Asian food and medicine for several millennia (Johnson et al., 2008). Species such as *Nostoc flagelliforme* Born. et Flah, *Spirulina platensis,* and *Nostoc sphaeroides* Kütz (NO) contain a wide range of bioactive compounds and have various functions (Chen, Juneau, & Qiu, 2007). *N. sphaeroides* Kütz, also known as Ge‐Xian‐Mi in China, has been used as a health product for centuries (Hao et al., 2011; Ku et al., 2013). *N. sphaeroides* Kütz is high in polysaccharide, amino acid, protein, vitamin, and mineral contents (Yang et al., 2011), and has bioactive properties, including antiviral, antitumor, antidiabetic, anti‐inflammatory, and lipid‐modulating activities (Ku, Kim, Pham, Yang, Wegner, et al., 2015; Ku, Kim, Pham, Yang, Weller, et al., 2015; Ku et al., 2013). Previous studies have shown that *N. sphaeroides* Kütz and its extracts significantly reduced plasma TC and TG levels in male C57BL/6j mice and may reduce atherosclerosis risk (Ku, Kim, Pham, Yang, Weller, et al., 2015; Yang, Kim, Park, & Lee, 2014). Nevertheless, the effects of *N. sphaeroides* Kütz on bodyweight and gut microbiota are unknown. Therefore, the understanding of the lipid‐lowering mechanisms and anti‐inflammatory effects of *N. sphaeroides* Kütz (NO) is a key to its application as a food supplement and in the food industry.

The present study aimed to explore the effects of NO supplementation on serum lipid levels, intestinal inflammation, integrity of the intestinal barrier, and gut microbiota associated with a HFD.

## 2 EXPERIMENT METHODS

### 2.1 Chemicals and reagents

*N. sphaeroides* Kütz (NO) used in this study was provided by Hunan Yandi Bioengineering Co., Ltd. Dry NO was crushed into powder at −20°C for 2 hr and then used directly in experiments. The main dry components of the NO used in the present research were fiber (47.3%), protein (30.8%), moisture (5.54%), and ash (5.7%); remaining components were vitamins and minerals. Components were identified by Societe Generale de Surveillance S.A. Assay kits of total cholesterol (TC), triglyceride (TG), high‐density lipoprotein cholesterol (HDL‐C), and low‐density lipoprotein cholesterol (LDL‐C) were acquired from Nanjing Jiancheng Bioengineering Institute. ELISA kits for tumor necrosis factor‐α (TNF‐α), interleukin‐1β (IL‐1β), interleukin‐6 (IL‐6), and interleukin‐10 (IL‐10) were pursed from Wuhan Huamei Biological Engineering co. Ltd. Total RNA extraction regent, SYBR Green Master primer, and oligo(dT)18 were obtained from Roche.

### 2.2 Animals

Forty C57BL/6j male mice were purchased from Beijing Vital River Laboratory Animal Technology Co., Ltd. at 6 weeks of age and were allowed to acclimate to their surroundings for 1 week. All mice were housed in an air‐conditioned room (temperature 22°C ± 2°C) with a relative humidity of 50%–60% and 12‐hr light/dark cycle.

### 2.3 Experimental protocols

All animal procedures were in accordance with the Animal Care and Use Committee of Beijing Union University. At the end of adaptive feeding for 1 week, mice were randomly divided into four groups (*n* = 10 in each group, one animal per cage): the control group, the model group, the low‐dose group, and high‐dose group. Control group animals received an AIN‐93M control diet, while other groups were fed a modified high‐fat diet (HFD) based on AIN‐93M for 4 weeks. During weeks 5–10, control group animals were maintained on a control diet, the model group was fed an HFD, the low‐dose group was fed an HFD supplemented with 2.5% NO (w/w, 2.5% NO), and the high‐dose group was fed with HFD supplemented with 7.5% NO (w/w, 7.5% NO). All feed was provided by Beijing Keao Xieli Feed Co., Ltd., (Beijing, China). Dietary compositions are shown in Table 1. At week 4, blood samples were collected by retro‐orbital bleeding and transferred into centrifuge tubes. Serum samples were obtained by centrifugation at 4°C and 4,000 r/min for 10 min, and then stored at −80°C for further analysis. At the end of the experiment, mice were fasted for 16 hr. The following day, animals were weighed and anesthetized by intraperitoneal injection of barbital at 9:00 a.m. Blood samples were collected by orbital vein puncture. Intestinal tissue was collected and stored at −80°C for quantitative real‐time reverse‐transcription PCR (qRT‐PCR).

**TABLE 1:** Composition of assay diets

Ingredient	Control diet		HFD		2.5% NO		7.5% NO	
	g	kcal	g	kcal	g	kcal	g	kcal
Cornstarch	465.7	1862.8	235.7	942.8	228.1	912.3	213.8	855.1
Casein	140	560	110	440	107.6	430.3	103.0	412.2
Dextrinized cornstarch	155	620	155	620	151.6	606.4	145.2	580.8
Sucrose	100	400	100	400	97.8	391.2	93.7	374.7
Soybean oil	40	360	40	360	39.1	352.1	37.5	337.2
Choline bitartrate	2.5	0	2.5	0	2.4	0.0	2.3	0.0
Fiber	50	0	50	0	48.9	0.0	46.8	0.0
Mineral mix	35	0	35	0	34.2	0.0	32.8	0.0
Vitamin mix	10	40	10	40	9.8	39.1	9.4	37.5
L‐Cysteine	1.8	7.2	1.8	7.2	1.8	7.0	1.7	6.7
Lard	0	0	150	1,350	146.7	1,320.3	140.5	1,264.6
Cholesterol	0	0	10	90	9.8	88.0	9.4	84.3
yolk	0	0	100	900	97.8	880.2	93.7	843.1
NO powder	0	0	0	0	24.4	30.1^a^	70.3	86.6^b^
Total	1,000	3,850	1,000	5,150	1,000	5,057.0	1,000	4,882.8

Note

1: Solka‐Floc cellulose. 2: AIN‐93 mineral mix. 3: AIN‐93 vitamin mix.

^a,b^ A total kilocalorie of NO was calculated based on the amounts of kilocalories in proteins (30.8%) as multiplying the amount of protein grams by four.

### 2.4 Biochemical analysis in serum

Serum lipid analysis was performed as described previously (Kang, Pichiah, Abinaya, Sohn, & Cha, 2016). Serum concentrations of TC, TG, HDL‐C, and LDL‐C were determined using commercial assay kits (Nanjing Jiancheng Bioengineering Institute). Serum concentrations TNF‐α, IL‐1β, IL‐6, and IL‐10 were determined using ELISA kits (Wuhan Huamei Bioengineering Institute). All assays were performed according to the manufacturer's instructions.

### 2.5 Quantitative real‐time reverse‐transcription PCR

Total RNA from mouse tissue was extracted using a total RNA extraction kit (Servicebio) according to the manufacturer's protocol. Two micrograms of total RNA samples were used to synthesize cDNA using a Revert Aid First‐Strand cDNA Synthesis Kit (Thermo Scientific). Quantitative real‐time reverse‐transcription PCR (qRT‐PCR) was performed in triplicate using SYBR Green and a LightCycler 480 Real‐Time PCR System (Roche Diagnostics). Each well was loaded with a 20 μl sample, containing 2.5 μl cDNA, 2.0 μl target primers, 8.0 μl water, and 12.5 μl Kapa SYBR Fast Master Mix. Hot‐start PCR was performed for 40 cycles. Each cycle consisted of denaturation for 15 s at 95°C, annealing for 30 s, and elongation for 30 s at 60°C. Roche LightCycler software (version 1.5.0, Roche Diagnostics) was used for data analysis. The results were analyzed using the 2^−ΔΔCt^ method of analysis. Mean expression levels for control group mice were set as 100%. The primers used are shown in Table 2.

**TABLE 2:** Primer pairs used for the real‐time quantitative PCR analysis

GenBank ID	Gene name		Primer sequence (5’ to 3’)
NM_007393.3	β‐actin	Forward	GTGACGTTGACATCCGTAAAGA
		Reverse	GTAACAGTCCGCCTAGAAGCAC
NM_001278601.1	TNF‐α	Forward	GCATCCAGCTTCAAATCTCGC
		Reverse	TGTTCATCTCGGAGCCTGTAGTG
NM_008361.4	IL‐1β	Forward	CCCTCACACTCACAAACCACC
		Reverse	CTTTGAGATCCATGCCGTTG
NM_031168.2	IL‐6	Forward	CCCCAATTTCCAATGCTCTCC
		Reverse	CGCACTAGGTTTGCCGAGTA
NM_010548.2	IL‐10	Forward	TTTAAGGGTTACTTGGGTTGCC
		Reverse	AATGCTCCTTGATTTCTGGGC
NM_021297.2	TLR4	Forward	TGAGGACTGGGTGAGAAATGAGC
		Reverse	CTGCCATGTTTGAGCAATCTCAT
NM_009045.4	NF‐kB	Forward	AAGCACAGATACCACCAAGACAC
		Reverse	CGCACTGCATTCAAGTCATAGTC
NM_009386.2	ZO‐1	Forward	CTGGGCAAGGGATAGGAGTG
		Reverse	CCATCTCTTGCTGCCAAACTATC
NM_016674.4	Claudin‐1	Forward	ATGTGGATGGCTGTCATTGGG
		Reverse	GGACAGGAGCAGGAAAGTAGGA
NM_008756.2	Occludin	Forward	GGAGGACTGGGTCAGGGAAT
		Reverse	CCGTCTGTCATAATCTCCCACC

### 2.6 Sequence processing and diversity analysis

Cecal fecal samples were snap‐frozen in liquid nitrogen before storage at −80°C. Fecal DNA was extracted using a DNA isolation kit (Servicebio). The extracted DNA was amplified using primers targeted to the V3‐V4 region of the bacterial 16S RNA gene (341F: CCTAYGGGRBGCASCAG; 806R: GGACTACNNGGGTATCTAAT). Sequencing was performed by Novogene Biotechnologies Inc. using an Illumina HiSeq 2500 sequencer. The raw reads were quality filtered by the UCHIME algorithm, and clean reads were used for analysis. Clean reads were clustered into operational taxonomic units (OTUs) according to the Ribosomal Database Project database based on 97% sequence similarity using Uparse software (version 7.0.1001). Alpha diversity analysis (Shannon index, Simpson index, and Good's coverage) was performed using Qiime software (version 1.9.1). Beta diversity analysis involved in principal coordinate analysis (PCoA) and nonmetric multidimensional scaling (NMDS) was calculated using R software according to the relative abundance of microbiota at the OTU level.

### 2.7 Short‐chain fatty acid analysis in mice cecal fecal

Cecal fecal samples were collected from each mouse and snap‐frozen in liquid nitrogen before storage at −80°C. The concentration of short‐chain fatty acids (SCFAs; acetic, propionic, and butyric acids) were measured by gas chromatography (GC). The GC instrument (FuLi 9720) was fitted with an FFAP column (30 m 0.25 mm 0.25 µm, Agilent) with a flame ionization detector. An internal standard was used crotonic acid.

### 2.8 Statistical analysis

Statistical analysis was conducted using SPSS software for windows (version 22). Data were assessed by one‐way ANOVA and Newman–Keuls pairwise comparison. *p* values <.05 were considered significant differences. All data from assays are shown as mean ± *SEM*.

## 3 RESULTS

### 3.1 Bodyweight and food intake

**FIGURE 1:**
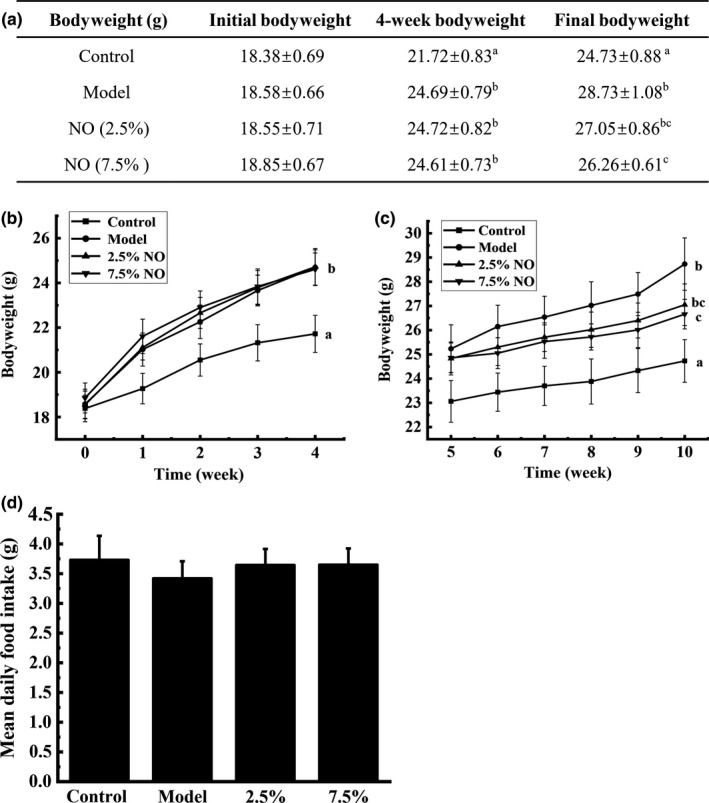
NO reduced bodyweight in HFD‐fed mice. (a) Animal bodyweight at different times, (b) effects of HFD on mice bodyweight, (c) effects of NO on bodyweight, and (d) daily food intake. Superscript marked with different letters represents statistically significant (*p* < .05). Values represent mean ± *SEM*; *n* = 10 in each group

Animal weights and food intake were monitored weekly. The initial bodyweights between groups were not significantly different. After 4 weeks of HFD, animals showed a significant increase (*p* < .05) in bodyweight compared with the control group (Figure 1a). Growth curves for the 10 weeks are shown in Figure 1b and c. It can be observed that HFD feeding for 10 weeks led to a significant increase in bodyweight (*p* < .05), while supplementation with NO prevented the bodyweight gain induced by HFD (Figure 1b and c). Mean daily food intake values are shown in Figure 1d. There was no significant difference among the four groups in daily food intake, indicating that the effects of NO on reducing bodyweight were not due to reduced food consumption.

### 3.2 NO ameliorated lipid metabolic disorders

The effects of NO on serum lipid levels, including TC, TG, HDL, and LDL, were investigated. The levels of serum lipid increased significantly *(p* < .05) after 4 weeks of the HFD, indicating that hyperlipidemia model animals were successfully built (Table 3). After 6 weeks of NO treatment, the levels of TC, TG, and LDL were less than model group, and HDL was higher in 7.5% NO group compared with model group (*p* < .05) (Table 4).

**TABLE 3:** Effects of HFD on lipid levels in serum

Group	Control	Model	2.5% NO	7.5% NO
TC (mmol/L)	3.12 ± 0.64^a^	8.29 ± 0.61^b^	8.49 ± 0.91^b^	8.62 ± 0.84^b^
TG (mmol/L)	1.01 ± 0.23^a^	1.87 ± 0.37^b^	1.89 ± 0.43^b^	1.97 ± 0.43^b^
HDL‐C (mmol/L)	1.87 ± 0.41^a^	1.45 ± 0.30^b^	1.41 ± 0.31^b^	1.14 ± 0.43^b^
LDL‐C (mmol/L)	0.51 ± 0.092	1.08 ± 0.17^b^	1.10 ± 0.17^b^	1.08 ± 0.11^b^

Note

Values represent mean ± *SEM*; *n* = 10 in each group. Superscript letters represent statistically significant differences (*p < *.05). Instances of the same letter between groups indicate that no statistically significant difference was found between the groups *(p > *.05).

**TABLE 4:** Effects of NO on lipid levels in serum

Group	Control	Model	2.5% NO	7.5% NO
TC (mmol/L)	3.96 ± 0.74^a^	7.65 ± 1.06^b^	5.93 ± 1.07^c^	5.77 ± 0.91 ^c^
TG (mmol/L)	0.71 ± 0.16 ^a^	1.81 ± 0.39^b^	1.2 ± 0.38 ^c^	1.03 ± 0.32^ac^
HDL‐C (mmol/L)	1.85 ± 0.39 ^a^	1.36 ± 0.35 ^b^	1.63 ± 0.29 ^c^	1.69 ± 0.25 ^c^
LDL –C (mmol/L)	0.63 ± 0.18 ^a^	1.24 ± 0.28 ^b^	1.09 ± 0.14 ^b^	0.98 ± 0.12 ^c^

Note

Values represent mean ± *SEM*; *n* = 10 in each group. Superscript letters represent statistically significant differences (*p < *.05). Instances of the same letter between groups indicate that no statistically significant difference was found between the groups *(p > *.05).

### 3.3 NO reduced inflammation in HFD‐fed mice

**FIGURE 2:**
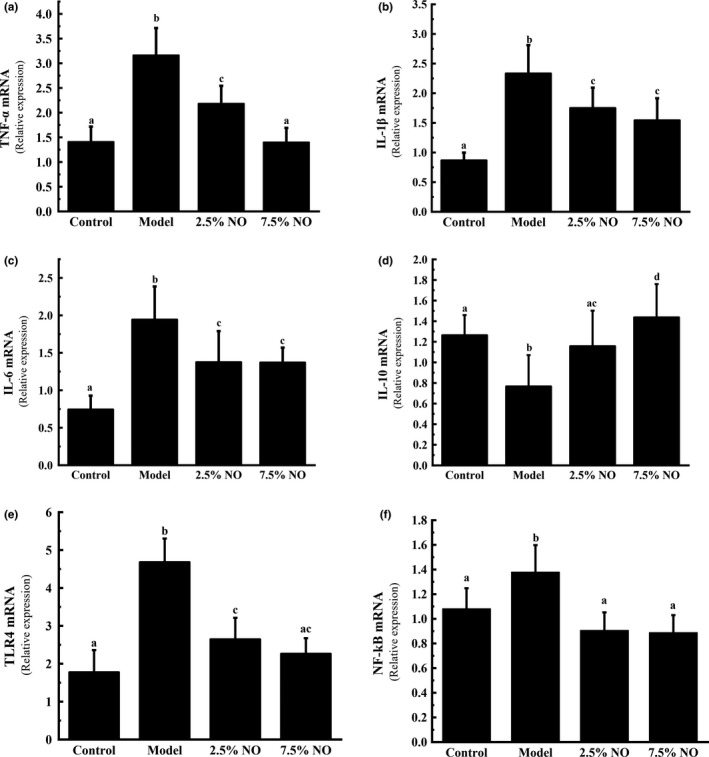
NO inhibited inflammation in mice intestine tissue. Relative mRNA expression levels of (a) TNF‐α, (b) IL‐1β, (c) IL‐6, (d) IL‐10, (e) NF‐kB, and (f) TLR4 in colon were assessed by using qRT‐PCR. Values represent mean ± *SEM*, *n* = 6 in each group. Letters represent statistically significant differences (*p* < .05). Instances of the same letter between groups indicate that no statistically significant difference was found between the groups (*p* > .05)

Studies have reported that HFD‐fed mice produce higher levels pro‐inflammatory cytokines, including TNF‐α, IL‐1β, and IL‐6, than those with a normal diet; in contrast, levels of the anti‐inflammatory cytokine IL‐10 are lower in HFD‐fed mice (Lu et al., 2016; Nam et al., 2018; Osborn & Olefsky, 2012). Serum TNF‐α, IL‐1β, IL‐6, and IL‐10 concentrations were measured in the present study. The mRNA expression of these cytokines was also measured in colon tissue. Serum TNF‐α, IL‐1β, and IL‐6 concentrations were higher in the model group mice, while IL‐10 was lower in the model group mice (*p* < .05) (Table 5). In 7/5% NO group, TNF‐α, IL‐1β, and IL‐6 expression levels were higher in model group animals, while IL‐10 expression level was lower (*p* < .05) (Figure 2a–f). Notably, the colon tissue mRNA expression levels of these cytokines were inversely correlated with the concentration of NO. The colon tissue expression levels of TNF‐α, IL‐1β, and IL‐6 were less than model group, and IL‐10 mRNA expression level was higher in the 2.5% and 7.5% NO group compared with the model group (*p* < .05). Expression levels of these cytokines in the 2.5% and 7.5% NO groups were closer to those of control group mice than HFD‐fed mice (Figure 2a–d).

**TABLE 5:** Effects of NO on inflammatory cytokines in serum

Group	Control	Model	2.5% NO	7.5% NO
TNF‐α (pg/mL)	274.03 ± 25.47^a^	434.09 ± 42.66^b^	390.97 ± 38.95^c^	305.26 ± 36.05^d^
IL‐1β (pg/mL)	247.27 ± 37.25^a^	427.46 ± 42.53^b^	402.88 ± 41.83^b^	318.16 ± 39.53^c^
IL‐6 (pg/mL)	26.07 ± 4.13^a^	44.21 ± 4.26^b^	37.34 ± 4.67^c^	29.79 ± 3.61^a^
IL‐10 (pg/mL)	501.13 ± 43.06^a^	258.26 ± 20.38^b^	355.76 ± 38.05^c^	490.97 ± 39.65^a^

Note

Values represent mean ± *SEM*; *n* = 10 in each group. Superscript letters represent statistically significant differences (*p < *.05). Instances of the same letter between groups indicate that no statistically significant difference was found between the groups *(p > *.05).

**FIGURE 3:**
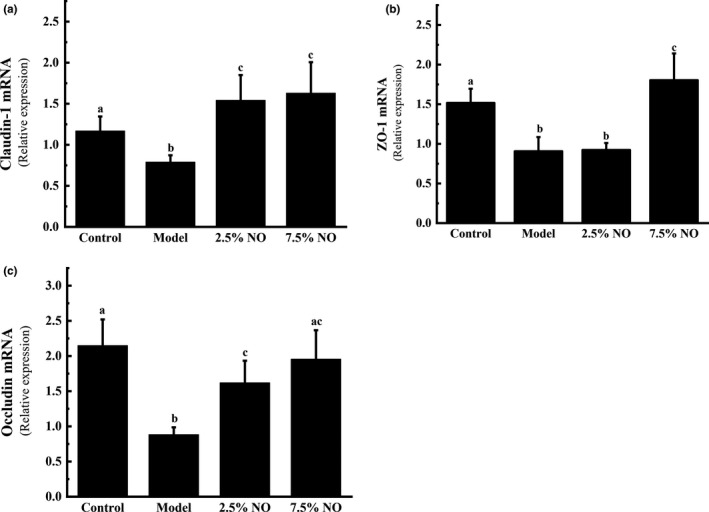
NO maintained intestinal integrity in HFD‐fed mice. Relative mRNA expression levels of (a) ZO‐1, (b) claudin‐1, and (c) occludin in intestine were assessed by using qRT‐PCR. Values represent mean ± *SEM*, *n* = 6 in each group. Letters represent statistically significant differences (*p* < .05). Instances of the same letter between groups indicate that no statistically significant difference was found between the groups (*p* > .05)

TLR4 signaling serves to initiate inflammation, controls the production of inflammatory cytokines in target tissues, and leads to chronic inflammation in HFD‐fed mice (Chan et al., 2016; Chang et al., 2015). TLR4 signaling pathways also initiate inflammation by modulating the activity of NF‐kB (Hoang et al., 2011). Therefore, the effects of NO on TLR4 and NF‐kB mRNA expression in colon tissue were examined in the present study. As shown in Figure 3e and f, the mRNA expression levels of TLR4 and NF‐kB were higher in model group animals compared with control group (*p* < .05). Mice treated with 2.5% and 7.5% NO had lower expression levels of TLR4 and NF‐kB than HFD mice without NO treatment (*p* < .05). Therefore, results suggest that NO reduced inflammation levels by modulating TLR4‐initiated NF‐kB signal cascades.

### 3.4 NO maintained intestinal integrity in HFD‐fed mice

Previous studies have shown that HFD may affect intestinal epithelial barrier permeability (Baye, Guyot, & Mouquet‐Rivier, 2017; Wu et al., 2017). We examined the mRNA expression of tight junction components involved in zonula occludens‐1 (ZO‐1), claudin‐1, and occludin in the mouse intestine. The mRNA expression levels of ZO‐1, claudin‐1, and occludin were less than other groups in model group animals (Figure 3a–c). This result is consistent with that of a previous study, which showed that HFD feeding may affect gut permeability (Monk et al., 2019). While protected by NO treatment, the mRNA expression of ZO‐1, claudin‐1, and occludin increased, especially in 7.5% NO group animals (*p* < .05) (Figure 3a–c). These results suggest that NO may have protective effects on intestinal barrier integrity in mice.

### 3.5 NO regulated HFD‐induced gut dysbiosis

**FIGURE 4:**
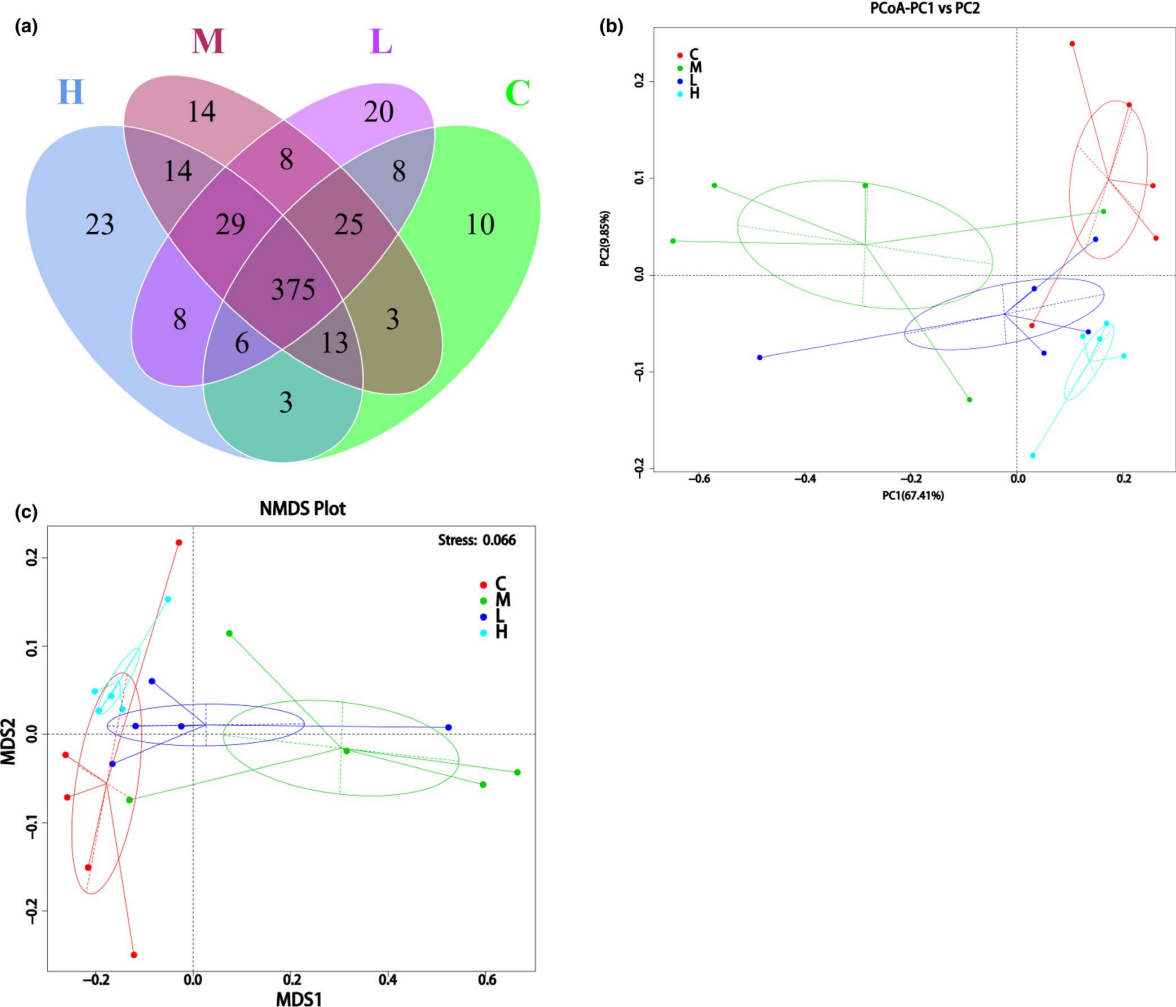
The impact of NO on the gut microbiota composition. (a) Venn diagram based on number of OTUs shared between different groups. Beta diversity showed by PCA (b) and NMDS (c) based on weighted UniFrac distance. C, control group; M, model group; L, 2.5% NO group; H, 7.5% NO group; *n* = 5 in each group

The effect of NO treatment on fecal microbiota structure was assessed by 16S rRNA (V3‐V4 region) sequencing of cecal fecal samples. A total of 1,464,817 raw reads were obtained. After selecting effective sequences, a total of 1,374,732 clean reads and an average of 68,736 ± 9,812 effective reads in each sample (*n* = 5 for each group) were generated. Shannon and Simpson index were used to analyze the sequencing diversity; the bacterial phylotype richness was reflected by ACE and Chao1 index. Species diversity and richness for each group are shown in Table 6. Sequencing diversity showed no significant difference between each group, while Chao1 and ACE index were much lower in the model group animals (Table 6). The degrees of OTUs shared in four groups are represented in Figure 3a. A total of 375 OTUs were common for all groups (Figure 4), while unique OTUs were also exhibited by each group (10 for the control group, 14 for the model group, 20 for the 2.5% NO group, and 23 for the 7.5% NO group, respectively), indicating greater OTU diversity in NO‐supplemented groups. Principal coordinate analysis (PCoA) and nonmetric multidimensional scaling (NMDS) based on weighted UniFrac distance revealed a clear clustering of microbiota composition for each group (Figure 4b and c). Both 2.5% and 7.5% NO groups aggregated closer to the control group than the model group (Figure 4b and c). Table 7 shows the results of Adonis analysis. From *R* ^2^ value and *p* value, we could conclude that the results of PCoA and NMDS are reliable.

**TABLE 6:** Alpha diversity in different groups

Group	Control	Model	2.5% NO	7.5% NO
Shannon	5.25 ± 0.29	4.68 ± 0.94	4.98 ± 0.65	4.78 ± 0.18
Simpson	0.94 ± 0.016	0.86 ± 0.11	0.91 ± 0.067	0.92 ± 0.01
Chao1	268.97 ± 21.95	177.56 ± 21.95^a^	250.31 ± 32.65	259.67 ± 19.81
ACE	275.50 ± 18.73^a^	210.60 ± 31.86^b^	256.60 ± 14.88^ab^	253.18 ± 32.64^ab^

Note

Values represent mean ± *SEM*, *n* = 5 in each group. The superscript different letters represent statistically significant (*p* < .05), whereas the same letter suggests that there are no statistically significant differences between groups (*p* > .05).

**TABLE 7:** Adonis analysis

Vs group	*R* ^2^	*p*
L‐H	.26066	.009
L‐M	.14946	.06
L‐C	.27722	.007
H‐M	.4321	.007
H‐C	.42253	.01
M‐C	.28704	.014

Note

*R* ^2^ value means the ratio of the group variance to the total variance. *p* value <.05 means that the reliability of this test is high.

**FIGURE 5:**
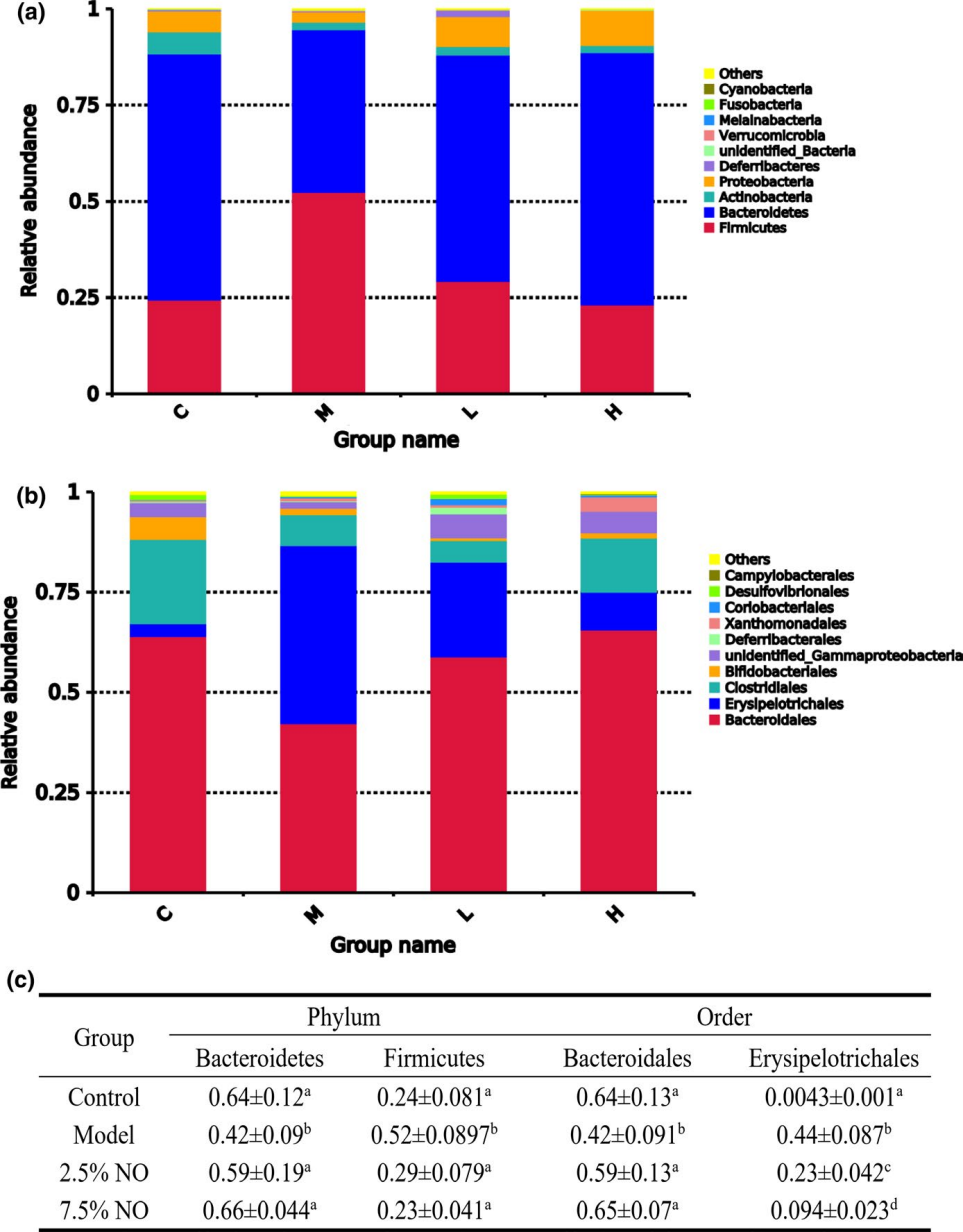
The impact of NO on the gut microbiota composition. (a) Relative abundance of bacteria at the phylum level and (b) relative abundance of bacteria at the order level. (c) Significant differences in statistical analysis in top 2. C, control group; M, model group; L, 2.5% NO group; H, 7.5% NO group; *n* = 5 in each group

Taxonomic analysis revealed the dominant species in gut microbiotas at different levels. Notably, at the phylum level, model group animals showed a higher level in Firmicutes and lower level in Bacteroidetes compared with other groups (Figure 5a) and showed a significant difference compared with other groups (Figure 5c). According to previous studies, the abundance of Firmicutes often showed higher level and Bacteroidetes lower level in HFD‐fed mice. Supplementation with NO reversed this situation (Figure 5a). At the order level, consistent with the phylum level, *Bacteroidales* showed a lower level in the model group, while NO groups showed higher level of Bacteroidales (Figure 5b). Erysipelotrichales are a class of Firmicutes; these showed higher in the model group, while the NO‐supplemented group exhibited lower levels of *Erysipelotrichales* in a dose‐dependent manner (Figure 5b). Figure 5c showed the statistical analysis of top two species at phylum and order level.

### 3.6 The effects of NO on cecal fecal SCFAs in HFD‐fed mice

**FIGURE 6:**
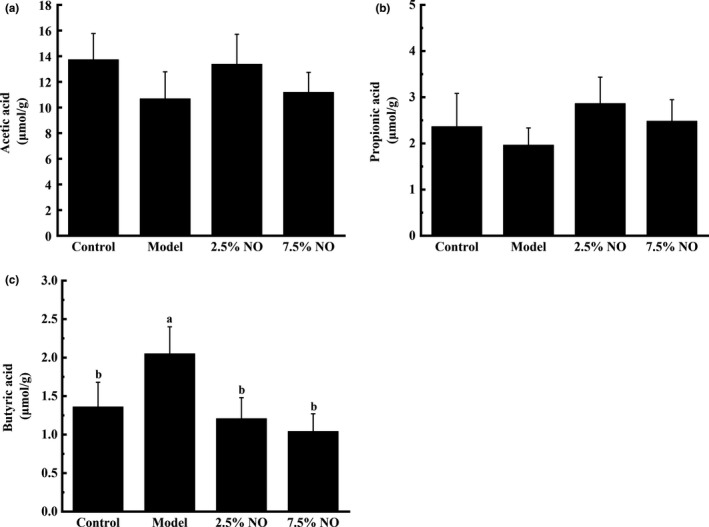
The effects of NO on cecal fecal SCFAs in HFD‐fed mice. Values represent mean ± *SEM* *n* = 8 in each group. (a) Acetic acids. (b) Propionic acids. (c) Butyric acids. Letters represent statistically significant differences (*p* < .05). Instances of the same letter between groups indicate that no statistically significant difference was found between the groups (*p* > .05)

The main SCFAs (acetic, propionic, and butyric acids) in cecal fecal were determined by gas chromatography. The concentrations of acetic and propionic acids showed no significant difference among the 4 groups (Figure 6a and b). The levels of butyric acids were higher in the model group animals compared with other groups (Figure 6c).

## 4 DISCUSSION

Although previous studies have reported that *N. sphaeroids kutz* lowered serum TC and TG and produced beneficial effects in hyperlipidemia mice, the effects of NO on the intestinal inflammation and gut microbiota have not been reported. In the present study, the effects of NO supplementation on HFD‐induced mice were assessed. Results showed that NO reversed dietary‐induced obesity, relieved intestinal inflammation, altered the gut microbiota, and protected intestinal integrity.

The current model of HFD‐induced chronic inflammation induced based on the dysbiosis of the gut microbiota and increased levels of blood lipopolysaccharide (LPS), also called metabolic endotoxemia (Cani et al., 2009). As seen in HFD‐fed animals, when the intestinal tube is in a state of dysbiosis it may gradually become leaky, allowing the LPS to enter enterohepatic circulation. Low blood concentrations of LPS blood may cause systemic and targeted inflammation in HFD‐fed mice through activation of TLR4 signaling in cells (Anhe et al., 2018). Results from the present study indicate that NO supplementation inhibited TLR4 gene expression and decreased inflammation in HFD‐fed mice. NO treatment also improved gut barrier integrity. The beneficial effects associated with NO treatment may therefore be attributed to specific alterations in the gut microbiota and the maintenance of gut barrier integrity.

The current study has shown that NO treatment led to remarkable changes in the gut microbiota composition, which partially improved the dysbiosis of microbiota induced by HFD. Results indicate that the gut microbiota can be changed by an HFD, and also modulated by dietary intervention. Therefore, NO may be used as a raw material in functional food to encourage gut microbiotas associated with reduced weight gain, inflammation, and disruption of the intestinal barrier in hyperlipidemia and obese animals. Previous studies have shown that the gut microbiotas of obese animals and humans are associated with increased levels of intestinal Firmicutes and decreased levels of Bacteroidetes, indicating that these two bacterial taxa may play an important role in hyperlipidemia in animals and humans (Makki, Deehan, Walter, & Backhed, 2018; Ojo et al., 2016; Schroeder & Backhed, 2016). A previous study suggested that feeding with plant hemicellulose can reverse a decrease in of Bacteroides‐to‐Firmicutes ratio and lead to weight loss (Koh, De Vadder, Kovatcheva‐Datchary, & Backhed, 2016). In the present study, NO supplementation in HFD‐fed mice led to a greater Firmicutes‐to‐Bacteroidetes ratio compared to model group mice (Figure 5a and b). Bifidobacterium spp. was previously reported to reduce obesity, but were not detected in the current study (Mathur & Barlow, 2015). Furthermore, Akkermansia was found to ease intestinal inflammation in another study (Everard, 2013). However, Akkermansia were not detected in NO‐treated and model group mice in the present study. This observation suggests that NO may produce beneficial effects by altering the Firmicutes‐to‐Bacteroidetes ratio and altering the levels of other bacterial species related to lipid metabolism. The contents of acetic, propionic, butyric, and total acids were analyzed in the present study. The contents of acetic and propionic acids showed no significant differences among the 4 groups, while the levels of butyric showed higher level in the model group animals (Figure 6a–c). A previous study suggested that butyric acid is one of the main products of Bacteroidetes. In the present study, model group mice had the highest level of Bacteroidetes, so the butyric acid in the model group mice showed higher level. These results are consistent with a previous study, which showed that bacteria of the Firmicutes phyla mainly produce butyric acid and Bacteroides mainly produce acetic and propionic acids (Louis, Hold, & Flint, 2014).

## 5 CONCLUSION

Notably, results showed that NO produced significant beneficial effects in HFD‐fed mice. Collectively, the results suggest that NO may be used as an ingredient for functional foods to reduce bodyweight gain and chronic inflammation in hyperlipidemia and obese individuals.

## CONFLICTS OF INTEREST

The authors declare that they have no conflicts of interest.

## ETHICAL STATEMENT

The authors declare that there is no conflict of interests. All experiments were conducted in accordance with “The Instructive Notions with Respect to Caring for Laboratory Animals” issued by the Ministry of Science and Technology of the People's Republic of China. The study's protocols and procedures were ethically reviewed and approved by Beijing Union University animal experiment ethics review committee.
